# Giant intracardiac thrombus-in-transit in a woman with sudden respiratory-haemodynamic compromise following cesarean section for eclampsia: a case report

**DOI:** 10.1093/ehjcr/ytaf145

**Published:** 2025-03-26

**Authors:** Olga Morelli, Dan M Gorfil, Yaron D Barac, Yaron Shapira, Alon Shechter

**Affiliations:** Department of Cardiology, Rabin Medical Center, 39 Jabotinsky St, Petach Tikva 4941492, Israel; Faculty of Medicine, Tel Aviv University, 35 Klachkin St, Tel Aviv 6997801, Israel; Faculty of Medicine, Tel Aviv University, 35 Klachkin St, Tel Aviv 6997801, Israel; Department of Cardio-Thoracic Surgery, Rabin Medical Center, 39 Jabotinsky St, Petach Tikva 4941492, Israel; Faculty of Medicine, Tel Aviv University, 35 Klachkin St, Tel Aviv 6997801, Israel; Department of Cardio-Thoracic Surgery, Rabin Medical Center, 39 Jabotinsky St, Petach Tikva 4941492, Israel; Department of Cardiology, Rabin Medical Center, 39 Jabotinsky St, Petach Tikva 4941492, Israel; Faculty of Medicine, Tel Aviv University, 35 Klachkin St, Tel Aviv 6997801, Israel; Department of Cardiology, Rabin Medical Center, 39 Jabotinsky St, Petach Tikva 4941492, Israel; Faculty of Medicine, Tel Aviv University, 35 Klachkin St, Tel Aviv 6997801, Israel

**Keywords:** Case report, Patent foramen ovale, Pulmonary embolism, Thrombectomy, Thrombosis, Thrombus-in-transit

## Abstract

**Background:**

Intracardiac thrombus-in-transit is a potentially fatal condition, seldom detected in real time.

**Case summary:**

We present a case of a 30-year-old pregnant woman with thalassemia intermedia and asplenia, who experienced a combined respiratory-haemodynamic collapse following an emergent caesarean section performed for eclampsia, and in whom a large mass transversing a patent foramen ovale was observed on bedside echocardiography. In view of the patient’s unstable condition, mass’ size and location as well as accompanying inter-atrial communication—all of which contributed to an imminent threat to cerebral circulation—and temporal proximity to abdominal surgery and epidural anaesthesia, an immediate open-heart surgery was decided upon that included mass excision and patent foramen ovale closure, and after which the patient quickly and fully recovered. Histopathologic examination of the mass revealed a mixture of thrombotic and amniotic fluid elements. Acute pulmonary embolism was eventually confirmed by computed tomography performed on post-operative day 2.

**Discussion:**

Early echocardiography and surgical intervention, as dictated by a multidisciplinary collaboration, allowed for a favourable outcome in our patient, emphasizing their pivotal role in the management of a life-threatening presentation of an intracardiac thrombus-in-transit.

**Learning points**

- Intracardiac thrombus-in-transit constitutes a rare medical emergency. When detected in the setting of a sudden respiratory and haemodynamic compromise, it usually signifies an ongoing pulmonary embolism.
- Bedside echocardiography is mandated in patients post–caesarean section experiencing haemodynamic collapse, and, in the case of an intracardiac thrombus-in-transit, serves for both diagnosis and devising treatment strategy.
- Prompt surgical intervention (i.e. open heart surgery) might be considered a life-saving procedure in unstable patients with an intracardiac thrombus-in-transit and holds the potential for full recovery.

## Introduction

Intracardiac thrombus-in-transit (TiT) refers to a noticeable, free-floating clot within the right heart chambers or venae cavae. Observed in ∼4% of patients with acute pulmonary embolism (PE), it is associated with a high risk of embolization and an above 40% mortality rate.^1, 2^ We herein report on a rather obscure case of an intracardiac TiT which, following prompt multidisciplinary response, was concluded with a favourable outcome.

## Summary figure

**Figure:**
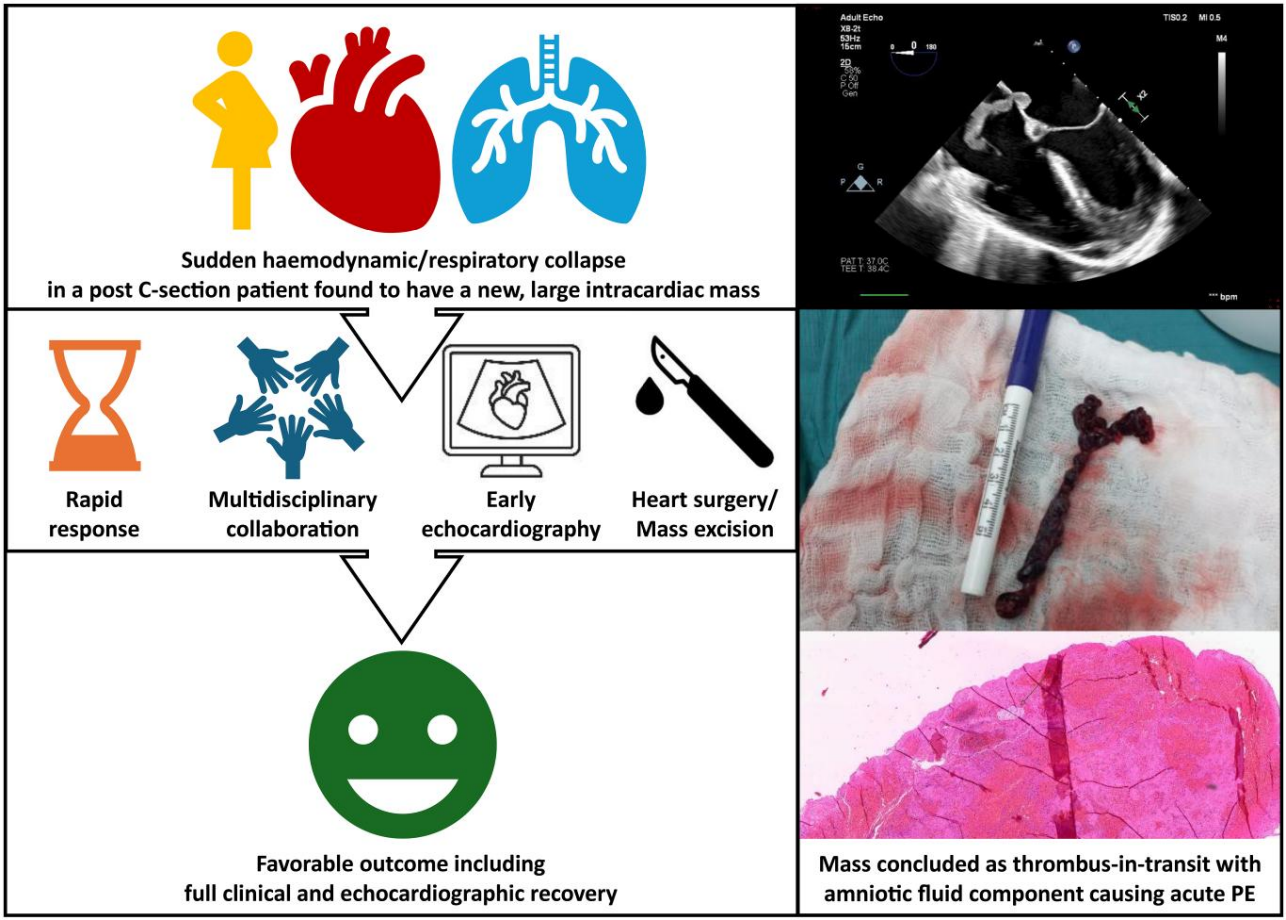


## Case presentation

A 30-year-old, gravida 4 para 3 woman with an otherwise uneventful pregnancy presented to the obstetric emergency department at 37 + 6 weeks gestation due to uterine contractions and a headache. Her medical history included thalassemia intermedia, for which she had been treated with packed red blood cells transfusions every few weeks on a regular basis; moderate hepatic iron deposition on repeat imaging studies that was attributed to these transfusions, with no signs of concomitant cardiac involvement according to an echocardiogram and a cardiac magnetic resonance imaging from the preceding 3 months and 2 years, respectively; and traumatic splenectomy as a teen. Per haematologists’ recommendation, she received 1 mg/kg of low molecular heparin daily during pregnancies.

**Figure 1:**
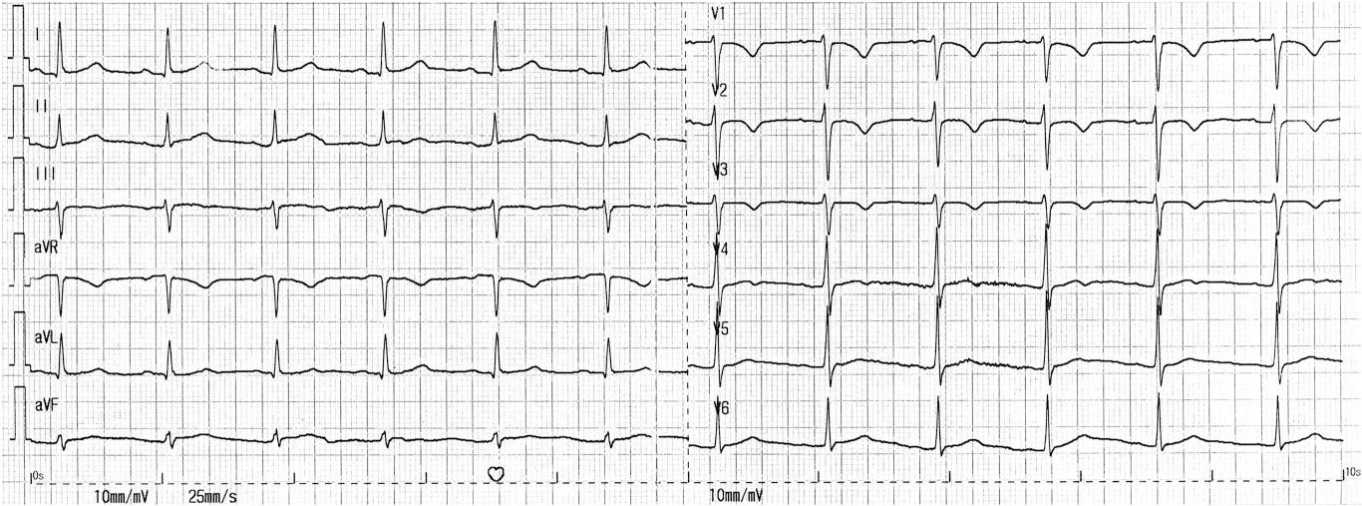
Initial electrocardiogram.

**Figure 2:**
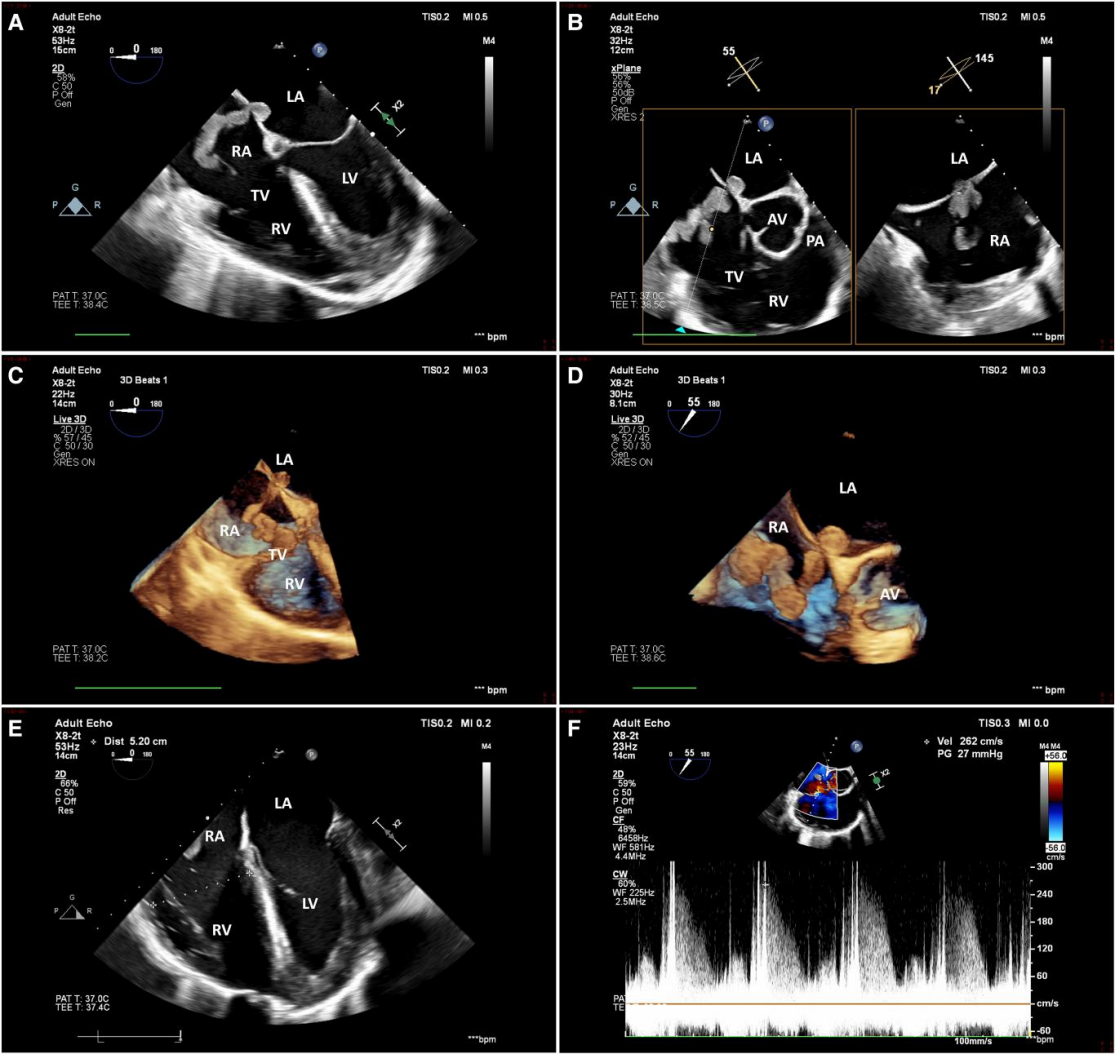
Initial transoesophageal echocardiogram. Initial transoesophageal echocardiogram revealed a large, sausage-like mass lodged in a patent foramen ovale that extended through the right atrium and intermittently protruded beyond the tricuspid valve and into a dilated, dysfunctional right ventricle. A mild-to-moderate tricuspid regurgitation with a peak pressure gradient of 27 mmHg were also demonstrated. *(A* and *E*) Oesophageal 4-chamber view, 2D; (*B* and *F*) oesophageal short axis view, biplane, 2D; (*C*) oesophageal 4-chamber view, live 3D; (*D*) oesophageal short axis view, live 3D. AV, aortic valve; LA, left atrium; LV, left ventricle; PA, pulmonic artery; RA, right atrium; RV, right ventricle; TV, tricuspid valve.

The patient’s examination was only notable for a systolic blood pressure of 180 mmHg, and a urine dipstick was positive for protein. A diagnosis of severe pre-eclampsia was made, and, accordingly, intravenous magnesium therapy and labour induction were ordered. During infusion, a short generalized, tonic-clonic seizure occurred, thus reframing the patient’s condition as overt eclampsia. As active labour was not yet achieved by that time, in terms of contractions frequency and cervical dilation, an emergent caesarean section was recommended. The surgery was performed under epidural anaesthesia, and a healthy male newborn was delivered. During suture of the fascia, the patient developed an acute respiratory distress, accompanied by desaturation and profound hypotension, and was therefore sedated, intubated, and placed on noradrenaline drip. Neither physical examination nor electrocardiography explained this sudden deterioration: neck veins were not distended, lungs were clear to auscultation, heart murmurs were not evident, lower extremities appeared normal, and there was a normal sinus rhythm with a non-specific T wave inversion in V1-V3 and without conduction anomalies (*Figure 1*). A point-of-care echocardiogram performed by the anaesthesiologist reportedly unveiled an unspecified echogenic lesion situated within a dilated, dysfunctional right ventricle. An urgent transoesophageal echocardiogram confirmed the presence of a large (9 over 1.5 cm), sausage-like mass lodged in a patent foramen ovale that, on one side, protruded into the left atrium and, on the other, extended through the right atrium and intermittently bulged beyond the tricuspid valve and into the right ventricle, the latter of which demonstrated a basal diameter of 5.2 cm, a fractional area change of 29%, and a subtle McConnell’s sign (*Figure 2*, Supplementary material online, *Videos S1*  *and*  *S2*). No additional masses nor significant left heart, valvular, or pericardial pathologies were noted. Specifically, there was a mild-to-moderate tricuspid regurgitation and the peak tricuspid regurgitation pressure gradient was 27 mmHg (*Figure 2*, Supplementary material online, *Video S3*).

A multidisciplinary team for managing the case was assembled that consisted of gynaecologists, anaesthesiologists, cardiologists, and cardiac surgeons. The general consensus was that the patient suffered an acute PE. Considering her chronic anticoagulation therapy, amniotic fluid embolism (AFE) was one explanation. Thrombus originating in the systemic venous system [i.e. deep venous thrombosis (DVT)] remained an additional possibility nonetheless, in view of several thromboembolic inciting factors, including thalassemia, pre-eclampsia/eclampsia, pregnancy, and surgery. An embolizing cardiac tumor was considered less likely in light of the recently normal imaging studies and general appearance of the mass.

**Figure 3:**
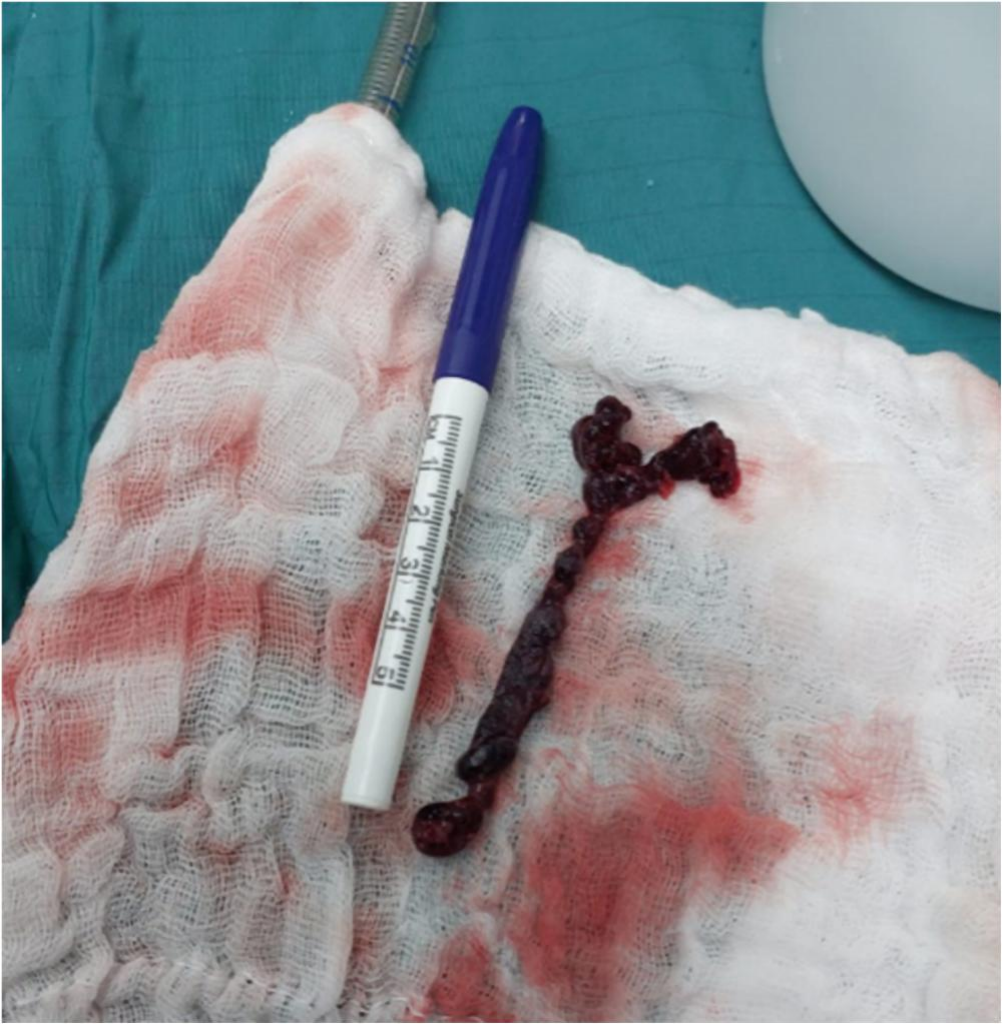
Gross macroscopic appearance of the intracardiac mass excised during surgery.

**Figure 4:**
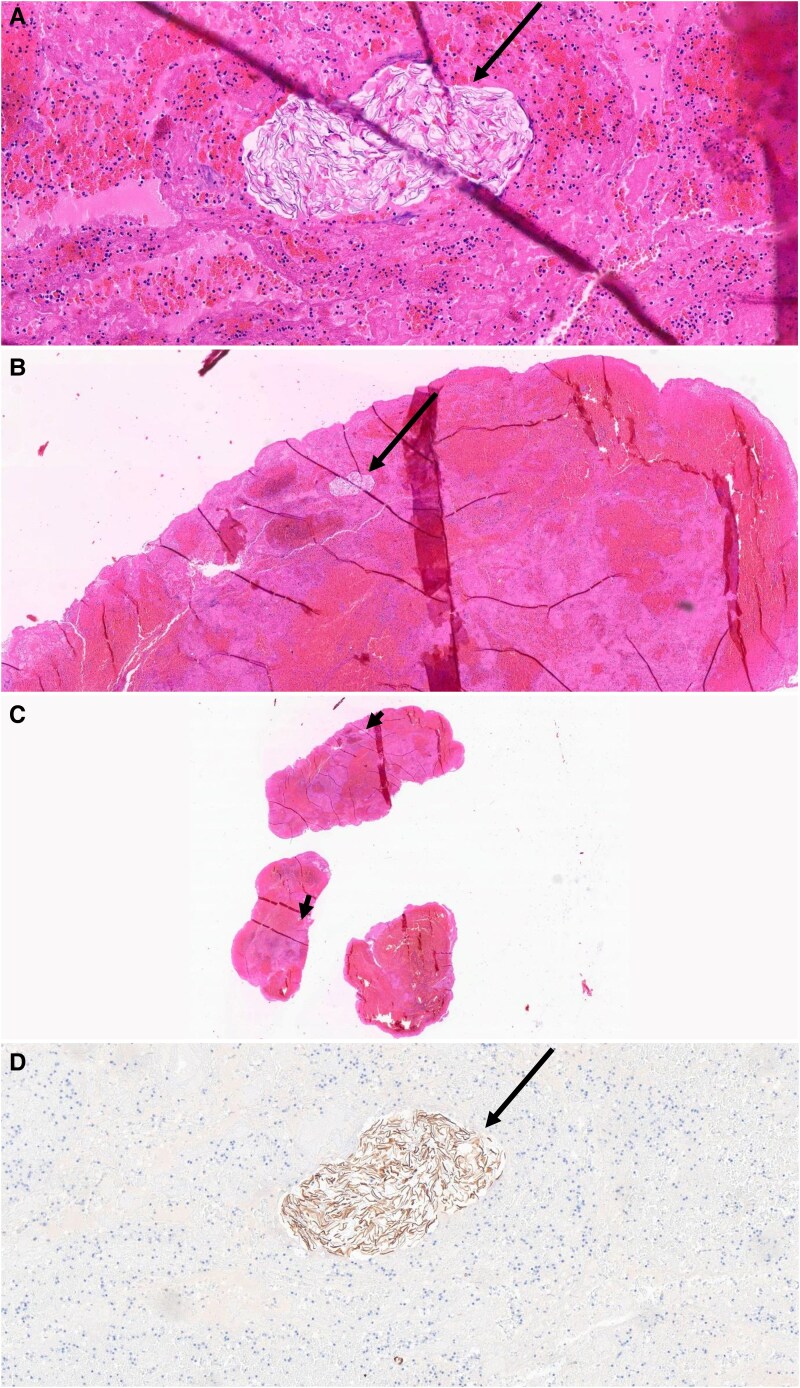
Histologic images of the intracardiac mass. Arrows (*A–D*) represent clusters of squamous cells, which stain positive (brown) for cytokeratin 5/6 in immunohistochemical image (*D*), amidst thrombotic material consisting of fibrin, erythrocytes, and inflammatory cells.

**Figure 5:**
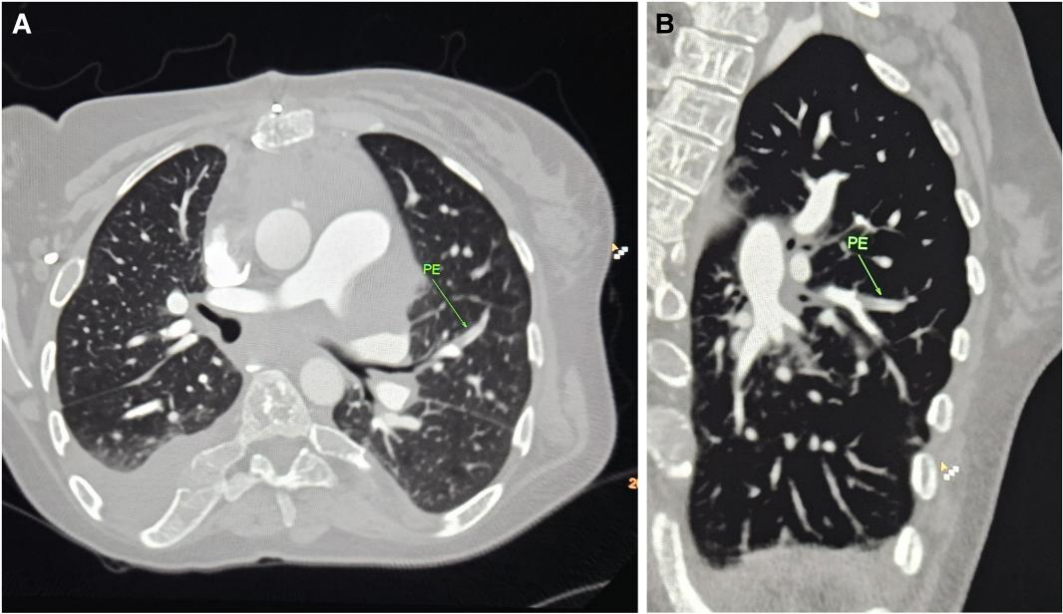
Chest computed tomography. PE, pulmonary embolus.

Due to the patient’s unstable condition, the immediate time proximity to abdominal surgery and epidural anaesthesia, the uncertainty regarding the nature of the mass, the latter’s protrusion into the left atrium from which it could jeopardize intracranial perfusion, and the availability of cardiothoracic team and operating room, a decision was made to proceed immediately to an open-heart surgery. Under direct vision and transoesophageal echocardiography monitoring, and while securing distal circulation by virtue of full cardiopulmonary bypass, a single mass was identified and evacuated in its entirety and the patent foramen ovale was sutured. Gross inspection of the lesion was consistent with a diagnosis of a thrombus (*Figure 3*). Histologic examination later suggested AFE features as well, manifested by clusters of squamous cells (*Figure 4*). Chest computed tomography performed on post-operative day (POD) 2 showed bilateral subsegmental PE (*Figure 5*). No obvious source for thromboembolism was discovered during workup that included lower limb ultrasound and extensive blood tests (see Supplementary material online, *Table S1*). Also, no clinical nor laboratory signs of consumption coagulopathy characteristic of classical AFE were evident. Brain computed tomography did not demonstrate embolic phenomena. Clinically, the patient improved dramatically following surgery and was weaned off vasoactive support within a few hours after the event and extubated on POD 2. Concurrently, and under full anticoagulation—which consisted of low molecular heparin for 5 days and later oral dabigatran—right ventricular function gradually recovered. On POD 4, the patient was transferred to the maternity unit and reunited with her newborn baby. By 2 months, she was back in her usual state. Considering the embolic event as a provoked one, the patient’s haematologists recommended full oral anticoagulation for 3–6 months and during future pregnancies. The patient was also advised against the use of oestrogen-based contraceptive pills.

## Discussion

A true medical emergency, intracardiac TiT carries a fierce prognosis; yet, evidence-based treatment approaches are lacking.^2–6^ Considered as the definitive therapy, surgical thrombectomy may prove life-saving in haemodynamically unstable patients.^7^ Further, it allows for addressing related conditions, such as an interatrial communication, which was observed in the current case. Percutaneous thrombus aspiration and percutaneous/systemic thrombolytic therapy may best be reserved for cases deemed unsuitable or too high risk for surgery, as both are associated with a heightened risk of failure and/or thrombus fragmentation with resultant distal embolization—especially in large and/or fixed masses such as the one presented here.^8,9^ Moreover, the two are somewhat contraindicated in the presence of recent pregnancy or surgery as well as epidural anaesthesia, all of which characterized our patient. Lastly, isolated systemic anticoagulation is less well-established in the treatment of large thrombi; is not applicable in life-threatening conditions due to a slow onset of action; and is problematic in the setting of an increased bleeding risk typical of the immediate post-surgery period.

In the case presented, a large intracardiac TiT was believed to have partially embolized to the lungs, causing a high-risk PE that manifested clinically with an acute respiratory and haemodynamic collapse following an emergent caesarean section. Perhaps due to its timing, well over 36 h after surgical thrombectomy and the commencement of anticoagulation therapy, chest computed tomography only demonstrated subsegmental lesions. The mechanism underlying the formation of the thrombus remained unclear and may have included a mixture of AFE and DVT. Caused by an abrupt break in the fetal–maternal barrier, AFE is characterized by the entrance of amniotic material into the maternal circulation, where it augments inflammatory and procoagulant pathways, culminating in multi-organ failure that may be out of proportion to imaging findings.^10^ Although supported by histologic preparation and elevated non-specific inflammatory blood biomarkers (e.g. sedimentation rate), AFE diagnosis was not fully established due to the macroscopic shape of the thrombus, lack of consumption coagulopathy, and alternative plausible diagnoses. As for DVT, while no discrete source was detected, the patient certainly experienced a prothrombotic state due to the combination of pre-existent thalassemia intermedia complicated by iron overload,^11^ super-imposed pre-eclampsia/eclampsia,^12^ pregnancy, and surgery. Furthermore, the mass’ elongated contour implied a deep venous origin. Notably, PE constitutes 20% of thrombotic phenomena among patients with thalassemia intermedia, in whom its risk is further amplified by the absence of a functional spleen.^11^ In both AFE and full-blown eclampsia, timely delivery constitutes the mainstay of treatment and may prevent complications in mother and baby.

## Conclusions

This case underscores the importance of rapid diagnosis and intervention, based upon immediate multidisciplinary response, in the rare occurrence of an intracardiac TiT complicating late pregnancy and delivery. Moreover, it provides support for the key role of early echocardiography and referral to surgery, whenever feasible, in the management of a life-threatening presentation.

## Lead author biography


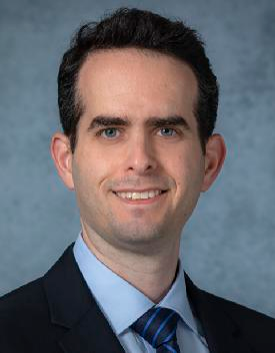


Dr Alon Shechter, MD MHA FESC graduated from the Tel Aviv University School of Medicine, and completed both his internal medicine residency and general cardiology fellowship at Rabin Medical Center. He later completed a clinical echocardiography fellowship at Rabin and a research fellowship at Cedars-Sinai Medical Center. Currently, Dr Shechter serves as a staff cardiologist at Rabin's echo lab and valve clinic. His research focuses on valvular heart disease and structural heart interventions.

## Supplementary Material

ytaf145_Supplementary_Data

## Data Availability

The full data underlying this article will be shared upon reasonable request to the corresponding author.

## Notes

**Consent:** Consent for publication has been obtained from the patient, in line with the COPE best practice guidelines.

## Notes

**Funding:** None.
