# Supplementary material for: Giant intracardiac thrombus-in-transit in a woman with sudden respiratory-haemodynamic compromise following cesarean section for eclampsia: a case report
Source: Eur Heart J Case Rep. 2025 Mar 26;9(4):ytaf145. doi: 10.1093/ehjcr/ytaf145 (PMC11971474; doi:10.1093/ehjcr/ytaf145)
Supplement: ytaf145_Supplementary_Data [file ytaf145_supplementary_data.zip › Supplement.docx]

**SUPPLEMENTARY MATERIAL**

**Table S1.** Relevant Blood Tests.

| **Parameter** | **Measured Value** | **Normal Reference Value** |
| --- | --- | --- |
| **Complete Blood Count** |  |  |
| Red Blood Cells (million/μL) | 3.25 | 4.10-5.10 |
| Hemoglobin Level (gr/dL) | 8.5 | 12.0-16.0 |
| Hematocrit (%) | 27.6 | 36.0-46.0 |
| Mean Corpuscular Volume (fL) | 84.9 | 78.0-102.0 |
| Reticulocyte Distribution Width (%) | 19.5 | 11.5-14.5 |
| Platelets (thousand/μL) | 744 | 150-450 |
| White blood cells (thousand/μL) | 14.51 | 0.50-11.00 |
| Neutrophils (%) | 95.0 | 40.0-74.0 |
| **Comprehensive Metabolic Panel** |  |  |
| Glucose Level (mg/dL) | 107 | 70-100 |
| Urea Level (mg/dL) | 22 | 17-43 |
| Creatinine Level (mg/dL) | 0.31 | 0.51-0.95 |
| Sodium Level (mEq/L) | 141 | 135-145 |
| Potassium Level (mEq/L) | 4.7 | 3.5-5.1 |
| Uric Acid Level (mg/dL) | 5.6 | 2.6-6.0 |
| Calcium Level (mg/dL) | 8.0 | 8.5-10.5 |
| Phosphorus Level (mg/dL) | 6.3 | 2.5-5.0 |
| Magnesium Level (mg/dL) | 2.04 | 1.60-2.60 |
| Total Protein Level (gr/dL) | 4.9 | 6.6-8.3 |
| Albumin Level (gr/dL) | 2.3 | 3.5-5.2 |
| Total Bilirubin Level (mg/dL) | 0.81 | 0.30-1.20 |
| Alkaline Phosphatase Level (IU/L) | 88 | 30-120 |
| Aspartate Aminotransferase Level (IU/L) | 130 | 0-31 |
| Alanine Aminotransferase Level (IU/L) | 53 | 0-34 |
| Gamma Glutamyl Transferase (IU/L) | 8 | 0-38 |
| Lactate Dehydrogenase Level (IU/L) | 531 | 135-214 |
| Amylase Level (IU/L) | 50 | 28-100 |
| Lipase Level (IU/L) | 19 | 16-63 |
| Total Cholesterol Level (mg/dL) | 130 | <200 |
| Triglycerides Level (mg/dL) | 249 | <150 |
| Low Density Lipoprotein Level (mg/dL) | 68 | <100 |
| High Density Lipoprotein Level (mg/dL) | 12 | >40 |
| **Coagulation Panel** |  |  |
| International Normalized Ratio | 0.95 | ≤1.10 |
| Prothrombin Time (sec) | 11.7 | 10.0-13.5 |
| Activated Partial Thromboplastin Time (sec) | 27.7 | 24.0-35.0 |
| Fibrinogen Level (mg/dL) | 545 | 200-530 |
| Lupus Anticoagulant Russell’s Viper Venom Time Ratio |  |  |
| Screen | 0.83 | 0.78-1.15 |
| Confirm | 0.94 | 0.86-1.00 |
| Lupus Anticoagulant Silica Clotting Time (sec) |  |  |
| Screen | 0.80 | 0.88-1.27 |
| Confirm | 1.02 | 0.80-1.20 |
| Antithrombin-III Activity (%) | 86 | 80-120 |
| Protein C Activity | 95 | 70-120 |
| Activated Protein C Resistance Ratio | 2.61 | >2.2 |
| Protein S Antigen Activity | 94.8 | 59.0-125.0 |
| Anti Cardiolipin IgA Antibody Level (IU/mL) | <2.0 | 0.0-19.9 |
| Anti β_2_ Glycoprotein Antibody Level (IU/mL) |  |  |
| IgA | <2.0 | 0.0-19.9 |
| IgG | <2.0 | 0.0-19.9 |
| IgM | <2.0 | 0.0-19.9 |
| Antinuclear Antibody Titer | Negative |  |
| Anti Double Stranded DNA Antibody Screen | Negative |  |
